# Four Waves of Hepatocyte Proliferation Linked with Three Waves of Hepatic Fat Accumulation during Partial Hepatectomy-Induced Liver Regeneration

**DOI:** 10.1371/journal.pone.0030675

**Published:** 2012-02-03

**Authors:** Yuhong Zou, Qi Bao, Sudhanshu Kumar, Min Hu, Guo-Ying Wang, Guoli Dai

**Affiliations:** Department of Biology, Center for Regenerative Biology and Medicine, School of Science, Indiana University-Purdue University Indianapolis, Indianapolis, Indiana, United States of America; National Cancer Institute, United States of America

## Abstract

Partial hepatectomy (PH) triggers hepatocyte proliferation–mediated liver repair and is widely used to study the mechanisms governing liver regeneration in mice. However, the dynamics of the hepatocyte proliferative response to PH remain unclear. We found that PH-induced mouse liver regrowth was driven by four consecutive waves of hepatocyte replication. The first wave exhibited the highest magnitude followed by two moderate waves and one minor wave. Underlying this continuous hepatocyte replication was persistent activation of cell cycle components throughout the period of liver regeneration. Hepatocyte mitotic activity in the first three proliferative cycles showed a circadian rhythm manifested by three corresponding mitosis peaks, which were always observed at Zeitgeber time 0. The Bmal1-Clock/Wee1/Cdc2 pathway has been proposed by others to govern the circadian rhythm of hepatocyte mitosis during liver regeneration. However, we did not observe the correlations in the expression or phosphorylation of these proteins in regenerating livers. Notably, Bmal1 protein displayed frequent changes in hepatic distribution and cellular localization as the liver regrowth progressed. Further, three waves of hepatic fat accumulation occurred during hepatic regeneration. The first started before and lasted through the first round of hepatocyte proliferation, whereas the second and third occurred concomitantly with the second and third mitotic peaks, respectively.

**Conclusion:**

PH-induced liver regeneration consists of four continuous waves of hepatocyte proliferation coupled with three waves of hepatic fat accumulation. Bmal1, Wee1, and Cdc2 may not form a pathway regulating the circadian rhythm of hepatocyte mitosis during liver regeneration.

## Introduction

The liver is innately able to regenerate in response to its mass loss caused by a variety of injuries. The initiation, expansion, and termination of the regenerative response are composed of numerous highly coordinated biological events that occur simultaneously or sequentially at the molecular, cellular, and tissue levels [1], [2], [3]. Central to these events is mature adult hepatocyte proliferation, as the first line of regenerative response to acute or early chronic liver injury [4], [5]. The initiation responses, including the production of priming factors (e.g., IL-6 and TNFα), are thought to prime hepatocytes to respond to growth factors (e.g., HGF and EGF) [5]. Subsequently, those growth factors stimulate quiescent hepatocytes to enter the cell cycle. Replicating hepatocytes elaborate a number of growth factors and cytokines to induce nonparenchymal cell proliferation [6]. Repopulation with newly generated liver cells is accommodated by massive tissue remodeling, eventually restoring the lost liver mass. Thus, hepatocyte proliferation constitutes the fundamental process and driving force of liver regrowth. Therefore, understanding the regulation of hepatocyte replication is of paramount importance in developing clinical approaches toward liver repair.

Two-thirds partial hepatectomy (PH) represents the most commonly used model for studying liver regeneration because it is not associated with massive necrosis or inflammatory complications [7], [8]. In rodents, PH triggers most residual hepatocytes to undergo what is thought to be two rounds of cell division, eventually leading to restoration of liver mass in 5–7 days [9]. In the mouse, the progression of the first round of hepatocyte replication in response to PH has been thoroughly characterized. The first round of DNA synthesis peaks at 36 hours following PH and is accompanied by temporary but prominent hepatic fat accumulation (steatosis) [1], [2], [10], [11]. Subsequently, hepatocyte mitosis occurs consistently during a time of day determined by the circadian clock [12]. However, the dynamics of hepatocyte proliferation and hepatic fat accumulation during the entire course of liver regeneration remain unclear, although the PH model is extensively used. Thus, the aim of this study is to address this issue. We found that PH induces four continuous waves of hepatocyte replication of which the first three waves exhibit a circadian rhythm and are linked with three waves of hepatic fat accumulation.

## Materials and Methods

### Mice and PH

Male mice (3 months old) on a C57BL6/129SV mixed background were used [13]. The mice were housed in plastic cages at 22±1°C on a 12-hour light/12-hour dark cycle with light on from 6:00 am to 6:00 pm. Standard rodent chow and water were provided *ad libitum* throughout the entire feeding period. Standard two-thirds liver resection was performed following the procedure described previously [11], [14]. The surgery was performed between 10:00 am and 12:00 pm to minimize potential variability in the progression of liver regeneration associated with surgery time and the circadian clock [12]. Mice were sacrificed at the indicated time points after surgery. Livers were immediately excised and weighed. Part of each liver was fixed in 10% formalin and embedded in paraffin to prepare liver sections. Meanwhile, part of each liver was embedded in Optimal Cutting Temperature Compound (Tissue Tek, Torrance, CA) and frozen in liquid nitrogen to prepare frozen liver sections. The remainder of each liver was frozen in liquid nitrogen and stored at −80°C until use. All of the animal experiments were conducted in accordance with the National Institutes of Health Guide for the Care and Use of Laboratory Animals. Protocols for the care and use of animals were approved by the Indiana University-Purdue University Indianapolis Animal Care and Use Committee (Approval ID: SC183R).

### Histology and Immunohistochemistry

Formalin-fixed and paraffin-embedded liver sections were subjected to Ki-67 immunostaining to visualize and count proliferating hepatocytes or were stained with hematoxylin and eosin to quantify mitotic figures in hepatocytes. Ki67-postive hepatocytes and mitotic figures were counted in five randomly chosen microscope fields per section at 200× and 100× magnifications, respectively. Primary antibodies against Ki-67 (Thermo Scientific, Fremont, CA) and Bmal1 (Santa Cruz Biotechnology, Santa Cruz, CA) were used for immunostaining according to the manufacturer's instructions. Oil Red O (Sigma Chemical Co., St. Louis, MO) staining was carried out on frozen liver sections to visualize intracellular lipid droplets.

### Western Blot Analysis

Liver homogenates (10 or 30 µg) were separated by polyacrylamide gel electrophoresis under reducing conditions. Proteins from the gels were electrophoretically transferred to polyvinylidene difluoride (PVDF) membranes. Antibodies against cyclin D1, cyclin B1, cyclin E (Cell Signaling Technology, Danvers, MA), cyclin A2 (Epitomic, Burlingame, CA), Bmal1, Wee1, p-Cdc2 p34 (Tyr 15), Cdc2 p34, and glyceraldehyde 3-phosphate dehydrogenase (GADPH) (Santa Cruz Biotechnology, Santa Cruz, CA) were used as probes. Immune complexes were detected using the enhanced chemiluminescence system (Pierce, Rockford, IL).

## Results

### PH-induced liver regeneration consists of four consecutive waves of hepatocyte proliferation

**Figure 1:**
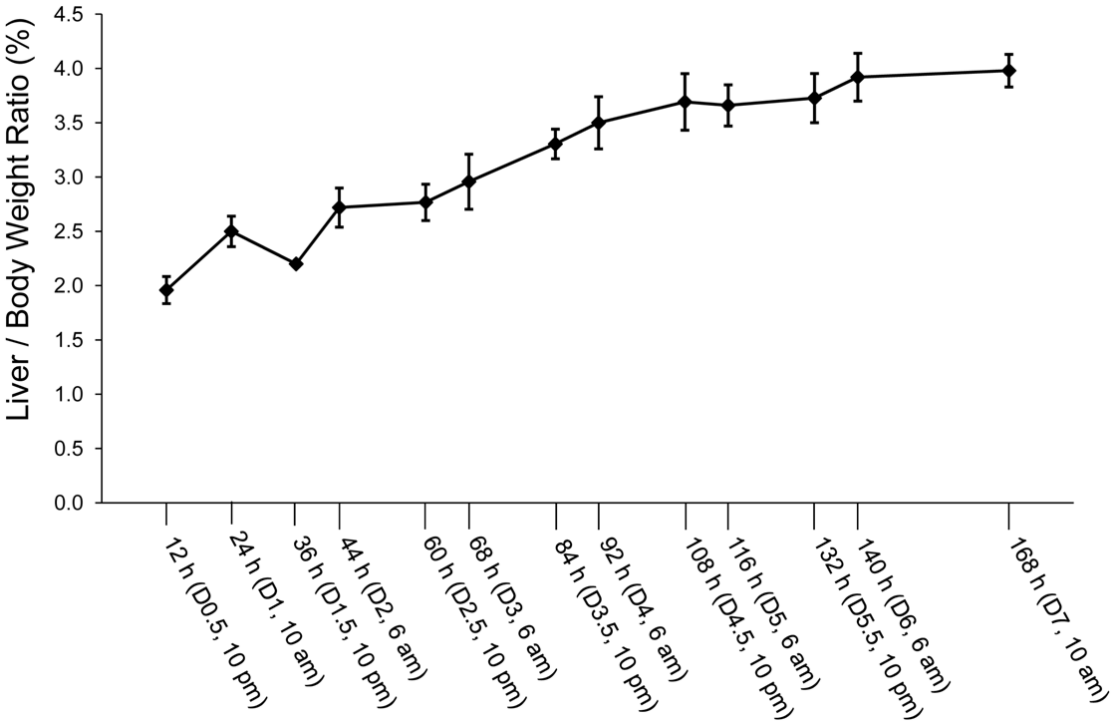
Liver regrowth following partial hepatectomy (PH). Mice were subjected to PH and sacrificed at the indicated time points. Liver and body weights were recorded. Liver-to-body weight ratio was used as a liver regrowth index. The results are presented as mean liver-to-body weight ratios ± SD (n = 3 mice per time point).

To determine the dynamics of hepatocyte proliferation during the entire period of liver regeneration, mice were subjected to PH and sacrificed at the time points presented in Figure 1. The ratio of liver weight to body weight at the time of sacrifice was used as a parameter to evaluate the progression of liver regrowth. As expected, robust liver regrowth took place during the first 6 days after PH (Fig. 1). At day 7 following PH, the liver-to-body weight ratio reached approximately 4%, implying that approximately 80% of the original liver mass was restored based on an average liver-to-body weight ratio of 4.9% in this strain of mice (Fig. 1). This pattern of PH-induced liver regrowth is consistent with the literature [8]. Among a total of 43 mice subjected to PH, the 4 mice that showed mobility reduction after surgery were sacrificed and were not included in the study.

**Figure 2:**
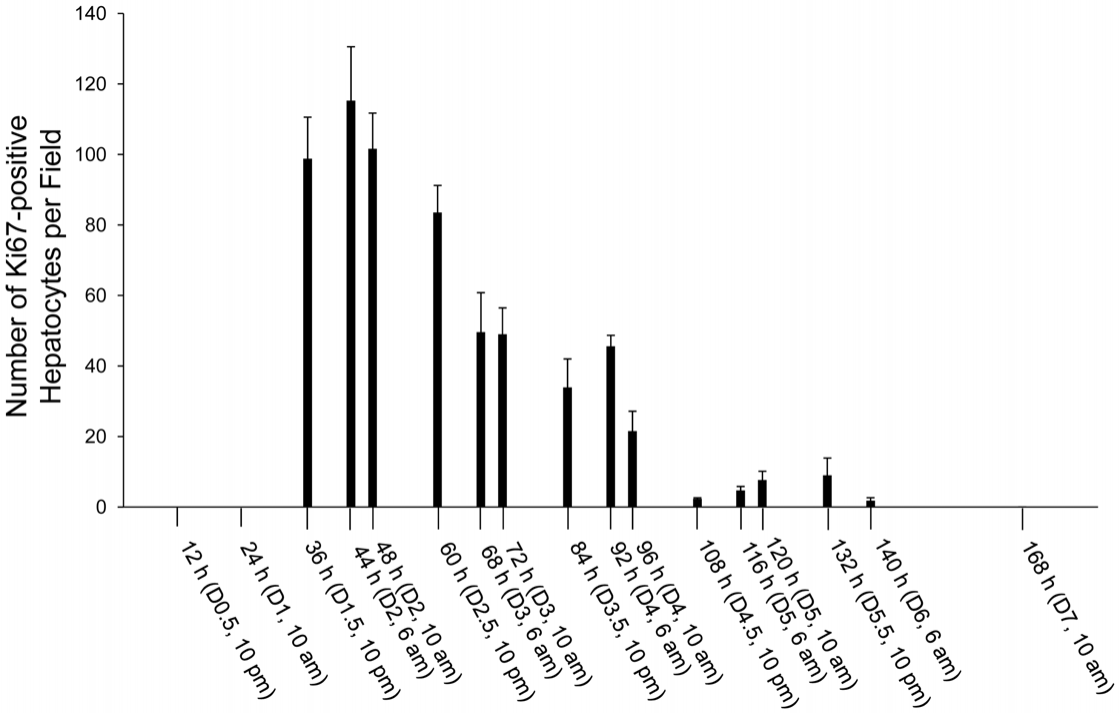
Hepatocyte proliferation induced by partial hepatectomy (PH). Mice were subjected to PH and sacrificed at the indicated time points. Ki-67 immunostaining was performed with formalin-fixed and paraffin-embedded liver sections. Ki67–positive hepatocytes were counted at 200× magnification in 5 randomly chosen fields per section. The results are shown as means per field ± SD (n = 3 mice per time point).

To estimate the numbers of proliferating hepatocytes at various stages of liver regeneration, Ki-67 immunostaining was performed on the sections prepared from regenerating livers at the time points indicated in Figure 2. Ki-67 protein is exclusively present in the nucleus during all active phases of the cell cycle and serves as a cell proliferation marker [15]. Increases in the numbers of Ki67-positive hepatocytes were seen during the first 6 days after PH (Fig. 2) in parallel with rapid liver regrowth during the same period (Fig. 1). Hepatocyte proliferation began after 24 h and was most robust in the first 2.5 days following PH. Subsequently, the number of replicating hepatocytes decreased by approximately half from day 3 to 4 after PH. Only a small population of hepatocytes underwent proliferation on day 4.5 and thereafter. Clearly, the number of proliferating hepatocytes was correlated with the extent of restoration of lost liver mass. These data support the notion that hepatocyte proliferation constitutes a fundamental cellular mechanism responsible for the hepatic regenerative response to loss of liver mass.

**Figure 3:**
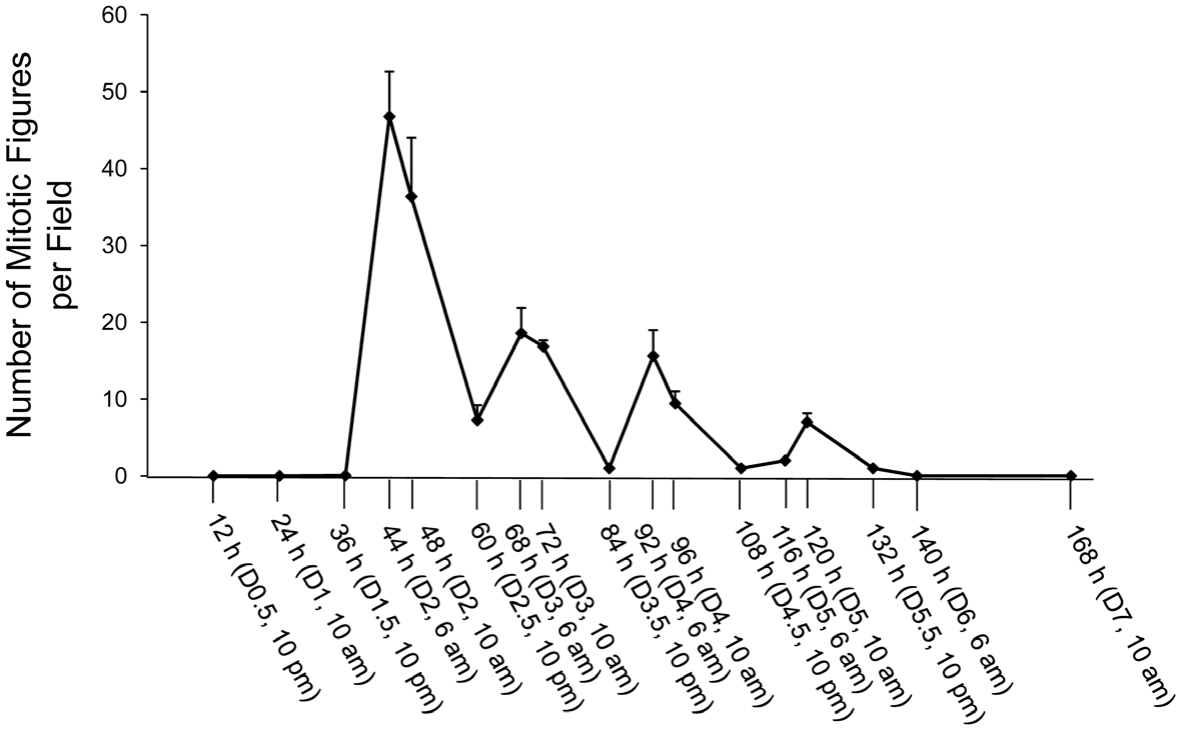
Dynamic of hepatocyte mitosis during partial hepatectomy (PH)-induced liver regeneration. Mice were subjected to PH and sacrificed at the indicated time points. Hepatic mitotic figures were counted at 100× magnification in 5 randomly chosen fields per liver section stained with hematoxylin and eosin. The data are shown as means per field ± SD (n = 3 mice per time point).

To gain insight into the progression of the cell cycle of proliferating hepatocytes, mitotic figures, representing hepatocytes undergoing mitosis, were quantified at the time points indicated in Figure 3. We found dynamic changes in the numbers of mitotic figures during the first 6 days post-PH (Fig. 3). The cyclicity of the increase and decrease in the number of mitotic figures revealed four waves of hepatocyte replication during the entire process of liver regeneration. The progression of the four rounds of the proliferative cycle exhibited several features. First, these four waves of hepatocyte replication occurred consecutively with an interval of approximately 24 hours between two adjacent mitosis peaks. Second, the number of hepatocytes going through each round of the cell cycle gradually reduced as the liver was restoring its lost mass. Additionally, the mitotic activity of hepatocytes entering the first three rounds of the cell cycle displayed a circadian rhythm, as mitosis was observed to peak only at 6:00 am (Zeitgeber time 0). These observations indicate that the number of hepatocytes entering into, and the timing of hepatocytes going through, each round of the cell cycle are highly regulated in regenerating livers.

**Figure 4:**
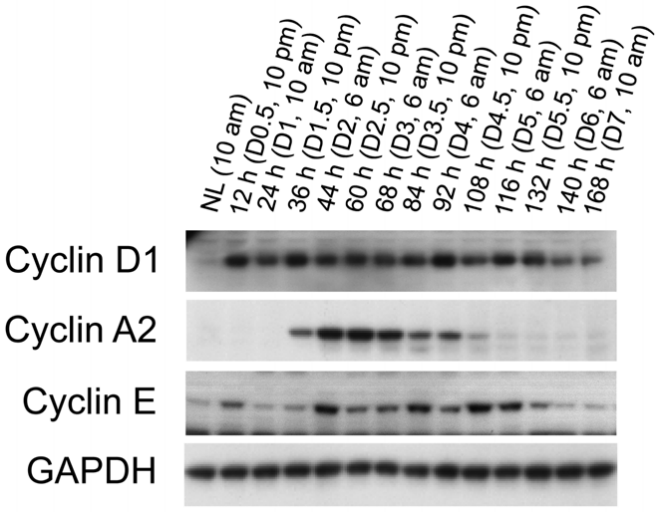
Hepatic expression of cell cycle components after partial hepatectomy (PH). Livers were collected at the indicated time points from normal mice and the mice subjected to PH. Liver lysates with equal amounts of protein from three mice per time point were pooled. Western blotting was performed with antibodies against the proteins indicated. Glyceraldehyde 3-phosphate dehydrogenase (GADPH) was used as a loading control. NL, normal liver.

To assess the progression of hepatocyte proliferation at the molecular level, the expression of major components of the cell cycle was analyzed with western blotting (Fig. 4). Cyclins D1 and E were activated as early as 12 h after PH, whereas cyclin A2 level increased at 36 h. Of note, the activation of these proteins persisted throughout the whole process of hepatocyte proliferation. These data indicate that persistent activation of cell cycle components underlies the four consecutive waves of hepatocyte replication during liver regeneration. Interestingly, the expression levels of these cell cycle components were not proportional to the magnitudes of hepatocyte replication waves. Cyclin D1 protein levels were consistently high throughout the first wave (from 24 to 60 hours after PH), the second wave (from 60 to 84 hours), the third wave (from 84 to 108 hours), and the fourth wave (from 108 to 132 hours) of the proliferative cycle. Although the magnitudes of the second and the third waves of hepatocyte replication were comparable, cyclin A2 protein showed wave-dependent expression levels. Cyclin E protein expression remained high in the weakest (the fourth) wave of hepatocyte proliferation, which lasted from 108 to 132 hours after PH. The wave-dependent and magnitude-independent expression levels of the cell cycle components suggest distinct mechanisms governing hepatocyte proliferation during the progression of liver regeneration.

### Bmal1, Wee1, and Cdc2 may not form a pathway controlling the circadian rhythm of hepatocyte mitosis during PH-induced liver regeneration

It has been proposed that the Bmal1-Clock/Wee1/Cdc2 pathway regulates the G2/M transition of the cell cycle of proliferating hepatocytes during liver regeneration [12]. Regardless of the time when PH is performed, hepatocyte mitosis always peaks at ZT0 (Zeitgeber time in a 12-hour light/12-hour dark cycle; ZT0 represents lights on, or 6:00 am in our animal facility). At that time, the levels of the circadian clock core components Bmal1 and Clock are low, leading to downregulation of their direct target gene Wee1, reduced phosphorylation of Wee1 substrate Cdc2 at Tyr 15, and, in turn, high activity of the Cdc2/cyclin B1 complex. As a result, hepatocytes stalled at G2 phase synchronously enter M phase, forming a peak of hepatocyte mitosis.

**Figure 5:**
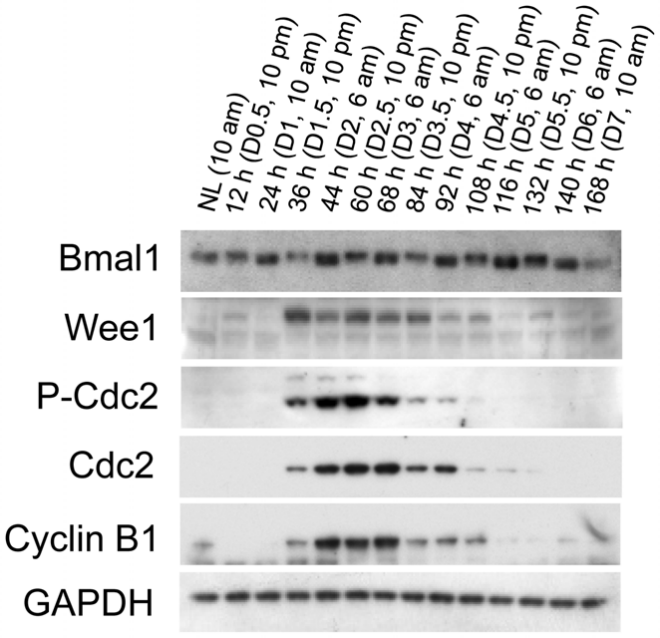
Protein expression of Bmal1, Wee1, Cdc2, and Cdc2 phosphorylated at Tyr 15 (p-Cdc2) in regenerating livers. Livers were collected at the indicated time points from normal mice and the mice subjected to PH. Liver lysates with equal amounts of protein from three mice per time point were pooled. Western blotting was performed with antibodies against the proteins indicated. Glyceraldehyde 3-phosphate dehydrogenase (GADPH) was used as a loading control. NL, normal liver.

To determine whether the Bmal1-Clock/Wee1/Cdc2 pathway is responsible for the circadian rhythm of hepatocyte mitosis in all four waves of hepatocyte replication, the components of this pathway were measured with western blotting (Fig. 5). Surprisingly, although the protein expression of Bmal1 showed a circadian pattern, the protein level of Bmal1 was always high at 6:00 am. Moreover, the rhythmic expression of Wee1 protein did not follow the circadian pattern of Bmal1 expression, because Wee1 was always low at 6:00 am. Furthermore, even though Cdc2 and cyclin B1 were activated during the same period as Wee1, Cdc2 was highly phosphorylated at Tyr 15 without exhibiting a circadian rhythm of phosphorylation compatible with Wee1 activity. Clearly, our data suggest that Bmal1, Wee1, and Cdc2 may not form a pathway regulating the circadian rhythm of hepatocyte mitosis during liver regeneration.

**Figure 6:**
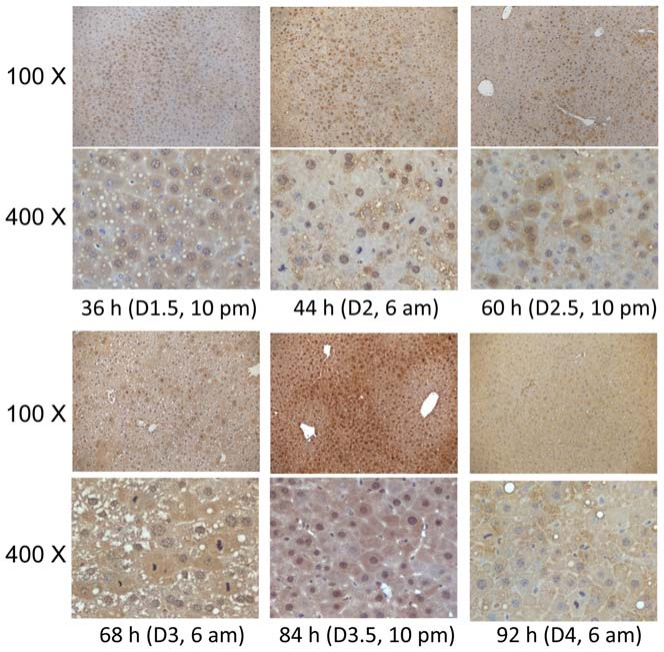
Distribution of Bmal1 protein in regenerating livers and hepatocytes. Bmal1 immunostaining was performed on liver sections prepared from formalin-fixed and paraffin-embedded hepatic tissues collected from mice sacrificed at the indicated time points after partial hepatectomy. 100× and 400× represent original magnifications.

To gain further insight into the behavior of Bmal1 in regenerating liver, Bmal1 immunostaining was performed with sections prepared from the livers collected at the indicated time points after PH (Fig. 6). The results show dynamic changes in Bmal1 protein distribution in the liver and in its localization within hepatocytes. On day 1.5 after PH, Bmal1 protein expression was restricted to certain areas of the liver sections and was detected in both the cytosol and nuclei of hepatocytes within those areas. On day 2, when the first peak of hepatocyte mitosis occurred, Bmal1 protein was widely expressed in the liver and existed predominantly in the nuclei of hepatocytes, suggesting a high transcriptional activity of the protein. On day 2.5, Bmal1 protein was detected unevenly in the liver and localized to both the cytosol and nuclei of positively stained hepatocytes. On day 3, when the second peak of hepatocyte mitosis took place, Bmal1 protein was observed throughout the hepatic tissue and in both the cytoplasm and nuclei of hepatocytes. On day 3.5, Bmal1 expression was concentrated in some areas of regenerating liver sections and localized in both the cytosol and nuclei of hepatocytes in those areas. On day 4, when the third wave of hepatocyte division peaked, Bmal1 protein exhibited even hepatic expression and mainly localized to the cytosol of hepatocytes. Therefore, as liver regeneration progressed, Bmal1 protein demonstrated a number of variations in distribution in the liver tissue and concomitantly within the hepatocytes themselves. Remarkably, within a vast majority of hepatocytes undergoing mitosis, Bmal1 protein was absent on day 2, abundant on day 3, and again absent on day 4. Taken together, these observations suggest complex temporal functions of Bmal1 during liver regeneration.

### Three waves of fat accumulation occur during PH-induced liver regeneration

**Figure 7:**
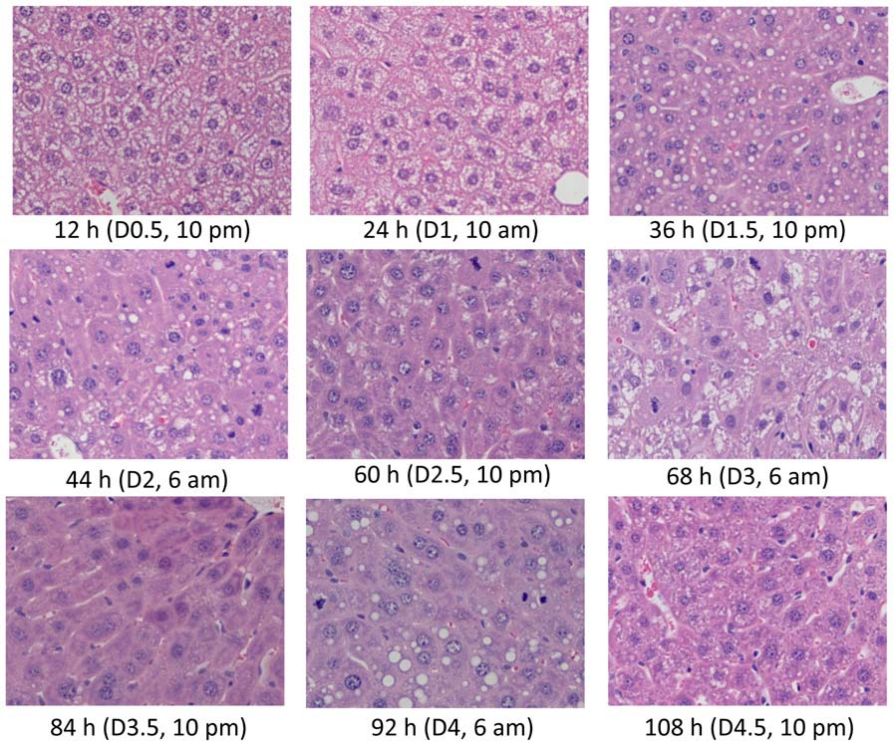
Histological analysis of regenerating livers. Livers were isolated at the indicated time points from mice that underwent partial hepatectomy. Formalin-fixed and paraffin-embedded liver sections were prepared and subjected to hematoxylin and eosin staining (400× original magnification).

**Figure 8:**
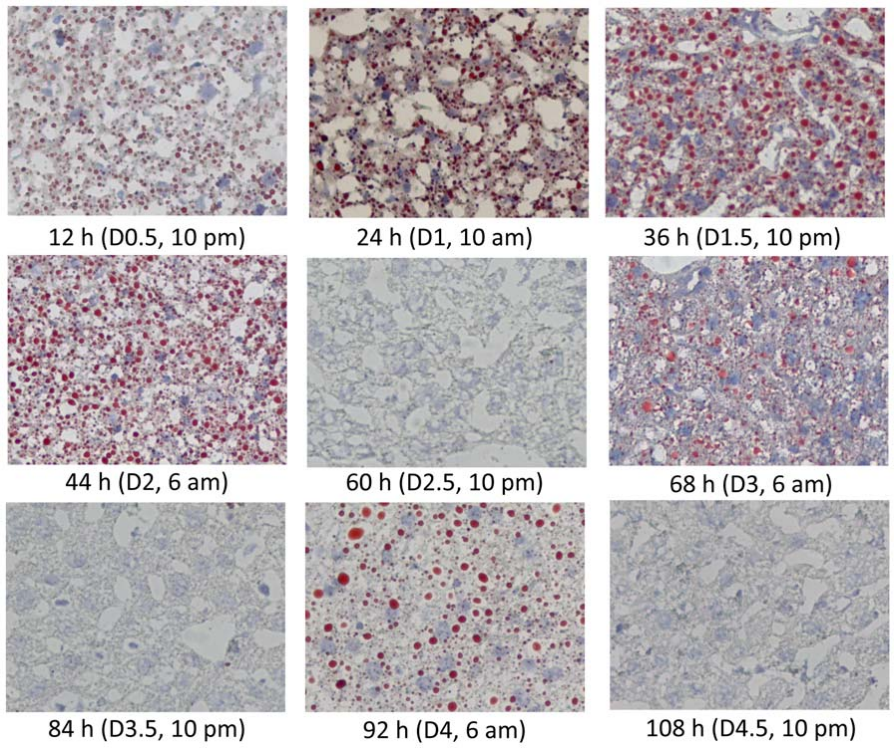
Hepatic fat accumulation in regenerating livers. Livers were isolated at the indicated time points from mice that underwent partial hepatectomy. Frozen liver sections were prepared and used for Oil red O staining (200× original magnification).

Temporary fat accumulation occurs during the first wave of hepatocyte proliferation (24 to 48 h after PH) as a prominent event associated with hepatocyte proliferation [16], [17]. To determine whether this event also takes place during the other three waves of hepatocyte proliferation, histological examination (Fig. 7) and Oil red O staining (Fig. 8) were performed on regenerating livers. We found that hepatic fat accumulation was evident as early as 12 h after PH, i.e., 12 h prior to the entry of hepatocytes into the first round of the cell cycle. As anticipated, fat droplets were seen at 24, 36, and 44 h following PH and throughout the period of the first wave of hepatocyte proliferation. Unexpectedly, another two waves of fat accumulation were observed at days 3 and 4 (6:00 am) after PH. Thus, fat accumulation occurred three times during liver regeneration. The first wave of fat accumulation took place prior to and during the first round, and the second and third waves occurred during the second and third rounds of hepatocyte proliferation. Apparently, hepatocyte replication and hepatic fat accumulation were coupled during liver regeneration.

## Discussion

To the best of our knowledge, this study is the first to report that four waves of hepatocyte proliferation drive PH-induced liver regeneration in the mouse. Our findings may provide insight into an innate fundamental program for regulating the dynamics of hepatocyte replication in response to liver mass loss. Based on our findings, we presume that in that program, two-thirds PH liver needs four continuous waves of hepatocyte replication to restore the deficit of hepatocytes. The number of hepatocytes entering each round of the cell cycle is tightly controlled. The first wave is the strongest, allowing the highest number of remaining hepatocytes to enter the cell cycle. This wave results in rapid expansion of the hepatocyte population to largely compensate for the lost metabolic capacity of injured liver. Immediately following are the second and the third waves, with fewer hepatocytes going through the cell cycle, and these waves are responsible for significant hepatic regrowth on the third and fourth days after PH. The combined number of replicating hepatocytes in the second and third waves is roughly identical to that in the first wave. The fourth wave is minor, serving as a fine-tuning step for the liver to precisely recover its original size. The driving force of these four waves of hepatocyte proliferation is the sustained activation of cell cycle components throughout the course of hepatic regeneration. In the regeneration program, the regulation of each round of the cell cycle is distinct, as evidenced by the fact that the expression levels of cyclins (D1, A2, E, and B1), p-Cdc2, and Cdc2 are independent of the magnitudes of the hepatocyte proliferation waves.

An increase of hepatocyte apoptosis has been seen by others via TUNEL assays at later stages (96 hours after PH) of liver regeneration in mice subjected to PH [18]. We evaluated the hepatic expression of intact and cleaved caspase 3 by western blotting in regenerating livers over the time course of 12 hours to 7 days post-PH. We found that cleaved caspase 3 was weakly detected only at day 5 after PH, although intact caspase 3 exhibited increased expression in regenerating livers throughout the time course studied (data not shown). This finding may suggest that liver cell apoptosis contributes somewhat to the fine-tuning of regenerated liver mass.

A majority of the studies using the PH model have focused on evaluating the scenario of the first wave of hepatocyte proliferation. In fact, the elimination of a single signal often delays or suppresses the first wave of hepatocyte replication. Eventually, liver regeneration can be completed, relying on compensatory effects derived from redundant signals [7]. Therefore, attention should be paid to the magnitudes of the other waves of the hepatocyte proliferative response to evaluate the effects of the elimination of the single signal on liver regeneration more precisely. In this regard, our findings can serve as a reference guide helping other investigators select appropriate time points after PH for their studies.

We found that the synchronization of hepatocyte mitotic activity in the first three proliferative cycles exhibited a circadian rhythm in regenerating liver. This finding is in line with the reports demonstrating that the circadian clock controls the timing of hepatocyte mitosis during liver regeneration [12], [19]. Matsuo et al. proposed that Wee1 is a link between the circadian clock and the cell cycle and that the Bmal1-Clock/Wee1/Cdc2 pathway governs the timing of hepatocyte division when the liver is regenerating [12]. However, as described above, our data do not support that proposed mechanism. The rhythmic expression of Wee1 protein neither followed the oscillation of Bmal1 protein expression nor led to a circadian pattern of phosphorylation of its substrate Cdc2 (Fig. 5). Moreover, the distribution of Bmal1 in hepatic tissue and its translocation within hepatocytes changed as liver regeneration proceeded (Fig. 6). Our study suggests that further study is required to determine how the circadian clock controls the progression of the cell cycle during the hepatic regenerative process. We noticed that there are several differences in the experimental design and approaches between our study and the work of Matsuo et al., including surgery times, time intervals for sample collection, and assay methods. It is difficult to compare the data directly, due to those differences. Nonetheless, we found that at the protein level, Bmal1, Wee1, and p-Cdc2 did not display a correlation that would support the mechanism proposed by Matsuo et al. linking that circadian pathway to hepatocyte mitosis during liver regeneration.

Interestingly, for the first time, we found that three waves of hepatic fat accumulation occurred during PH-induced liver regeneration. Obviously, these three waves of fat accumulation are coupled with the first three rounds of hepatocyte replication. Hepatic lipid accumulation after PH is a consequence of enhanced fatty acid mobilization and metastasis to the liver, as well as increased hepatic lipogenesis and decreased secretion of very-low-density lipoprotein [16], [17]. It has been proposed that hepatic lipid accumulation is required to meet the increased energy demand for rapid cell proliferation and is essential for the enhanced biosynthesis of membrane phospholipids during liver regeneration [20]. However, the conclusions from a number of studies on the role of hepatic fat accumulation during liver regeneration are controversial. Certain studies indicate that transient hepatic steatosis may be essential for efficient liver regeneration, whereas others show that altered hepatic lipid metabolism and accumulation did not interrupt the hepatic regenerative response. The discrepancy may be caused by the distinct approaches utilized by different investigators to manipulate lipid accumulation in regenerating liver, including dietary, pharmacological, and genetic means [21], [22], [23], [24], [25], [26], [27]. Therefore, the biological significance of PH-induced hepatic steatosis remains elusive. Our observations strongly suggest that this remarkable event is required for the hepatic regenerative response because it is linked with each major wave of hepatocyte proliferation.
